# LTP expression mediated by autonomous activity of GluN2B-bound CaMKII

**DOI:** 10.1016/j.celrep.2024.114866

**Published:** 2024-10-11

**Authors:** Nicole L. Rumian, C. Madison Barker, Matthew E. Larsen, Jonathan E. Tullis, Ronald K. Freund, Amir Taslimi, Steven J. Coultrap, Chandra L. Tucker, Mark L. Dell’Acqua, K. Ulrich Bayer

**Affiliations:** 1 Department of Pharmacology, University of Colorado Anschutz Medical Campus, Aurora, CO 80045, USA; 2 Program in Neuroscience, University of Colorado Anschutz Medical Campus, Aurora, CO 80045, USA; 3 These authors contributed equally; 4 Lead contact

## Abstract

Learning and memory are thought to require the induction and maintenance of long-term potentiation (LTP) of synaptic strength. LTP induction requires the Ca^2+^/calmodulin-dependent protein kinase II (CaMKII) but for structural rather than enzymatic functions. We show that the relevant structural function is regulated by CaMKII binding to the NMDA-type glutamate receptor subunit GluN2B. This binding directly generates Ca^2+^-independent autonomous CaMKII activity, and we show that this enzymatic activity is dispensable for LTP induction (within 5 min) but required for a subsequent LTP phase (within 15 min). This requirement for CaMKII activity provides an objective temporal definition for the intermediary phase of LTP expression. Later LTP maintenance may still require structural functions of GluN2B-bound CaMKII but not the resulting enzymatic CaMKII activity or their co-condensation. Thus, autonomous CaMKII activity mediates post-induction LTP but (1) via GluN2B binding, not T286 autophosphorylation, and (2) during the intermediary expression phase rather than for long-term maintenance.

## In brief

Rumian et al. demonstrate that enzymatic activity of CaMKII is not required for LTP induction or maintenance but is required for the intermediary phase of LTP expression. CaMKII pT286 was required only during LTP induction, whereas LTP expression was instead mediated by the autonomous activity of GluN2B-bound CaMKII.

## Graphical Abstract

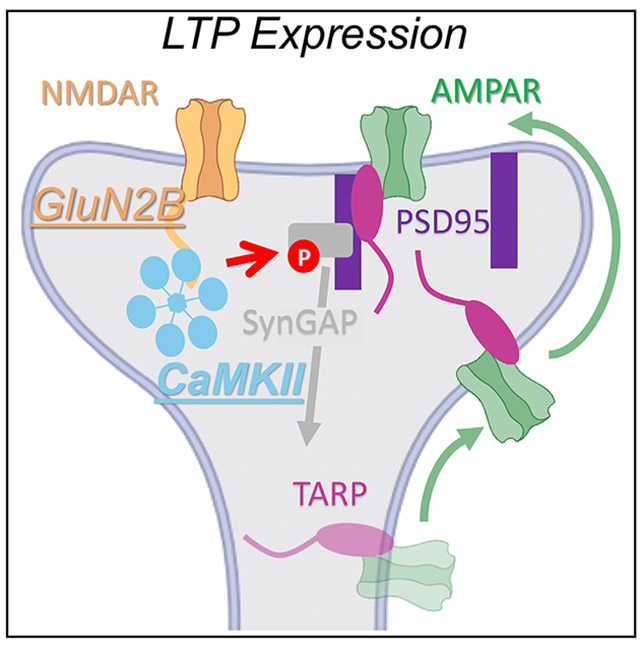

## INTRODUCTION

The Ca^2+^/calmodulin-dependent protein kinase II (CaMKII) has long been postulated to induce long-term potentiation (LTP) via its Ca^2+^-stimulated activity and then to maintain LTP, and thereby memory, via its Ca^2+^-independent “autonomous” activity that is induced by its autophosphorylation at T286 (pT286) (reviewed in Lisman et al.,^1,2^ Bayer and Schulman,^3^ Coultrap and Bayer,^4^ and Yasuda et al.^5^). However, recent evidence indicates that even LTP induction requires structural CaMKII functions rather than enzymatic activity.^6,7^ The only exception was the enzymatic activity required to induce pT286, which was proposed to be required to promote binding to the NMDA-type glutamate receptor (NMDAR) subunit GluN2B, which in turn was proposed to be among the structural CaMKII functions required for LTP.^6,8^ This suggested that neither LTP induction nor maintenance requires any other enzymatic CaMKII activity at all. However, this is not necessarily the case, as a potential role of enzymatic CaMKII activity in LTP maintenance could not be conclusively tested using previous pharmacological inhibitors^9^: CaM-competitive CaMKII inhibitors such as KN62 or KN93 do not interfere with Ca^2+^-independent autonomous CaMKII activity^10,11^ that is generated by T286 autophosporylation^12^ or GluN2B binding.^13^ Peptide inhibitors related to either AC3-I or CN21 all bind to the T-site on CaMKII, which is also the binding site for GluN2B,^13-15^ suggesting that CaMKII binding to these inhibitors and GluN2B could be mutually exclusive (although we show here that tatCN21 can inhibit activity of GluN2B-bound CaMKII). Accordingly, the effects of these peptides on LTP maintenance were mixed,^16-19^ with LTP reversal only seen with high concentrations that also disrupt CaMKII binding to the NMDAR^19^ (or when the effect on CaMKII binding was not tested^18^). Notably, it was shown 23 years ago that GluN2B binding directly maintains CaMKII in an active conformation,^13^ but a specific function of this activity has remained elusive. More recently, CaMKII has also been described to interact with GluN2B via liquid-liquid phase separation (LLPS),^20,21^ a form of biomolecular condensation that has gained traction as a potential organizing principle at excitatory synapses.^22^ In contrast to traditional binding that is mediated by a single high-affinity interaction, condensation is generally mediated by multiple low-affinity interactions. Here, we used a combination of genetic mutations and different CaMKII inhibitors (including various inhibitors that can block the activity of GluN2B-bound CaMKII) to show that (1) GluN2B binding is indeed the structural CaMKII function required for LTP induction, whereas (2) the enzymatic activity of this GluN2B-bound CaMKII is required during LTP expression, with (3) no requirement for pT286 beyond the initial induction phase, (4) no requirement for any enzymatic CaMKII activity during LTP maintenance beyond the first 15 min, and (5) the potential for a structural function of the traditional CaMKII binding to GluN2B but not of their co-condensation during the maintenance phase.

## RESULTS

### Rescue of LTP induction in T286A mutant slices by AS397 or ruxolitinib

**Figure 1.**
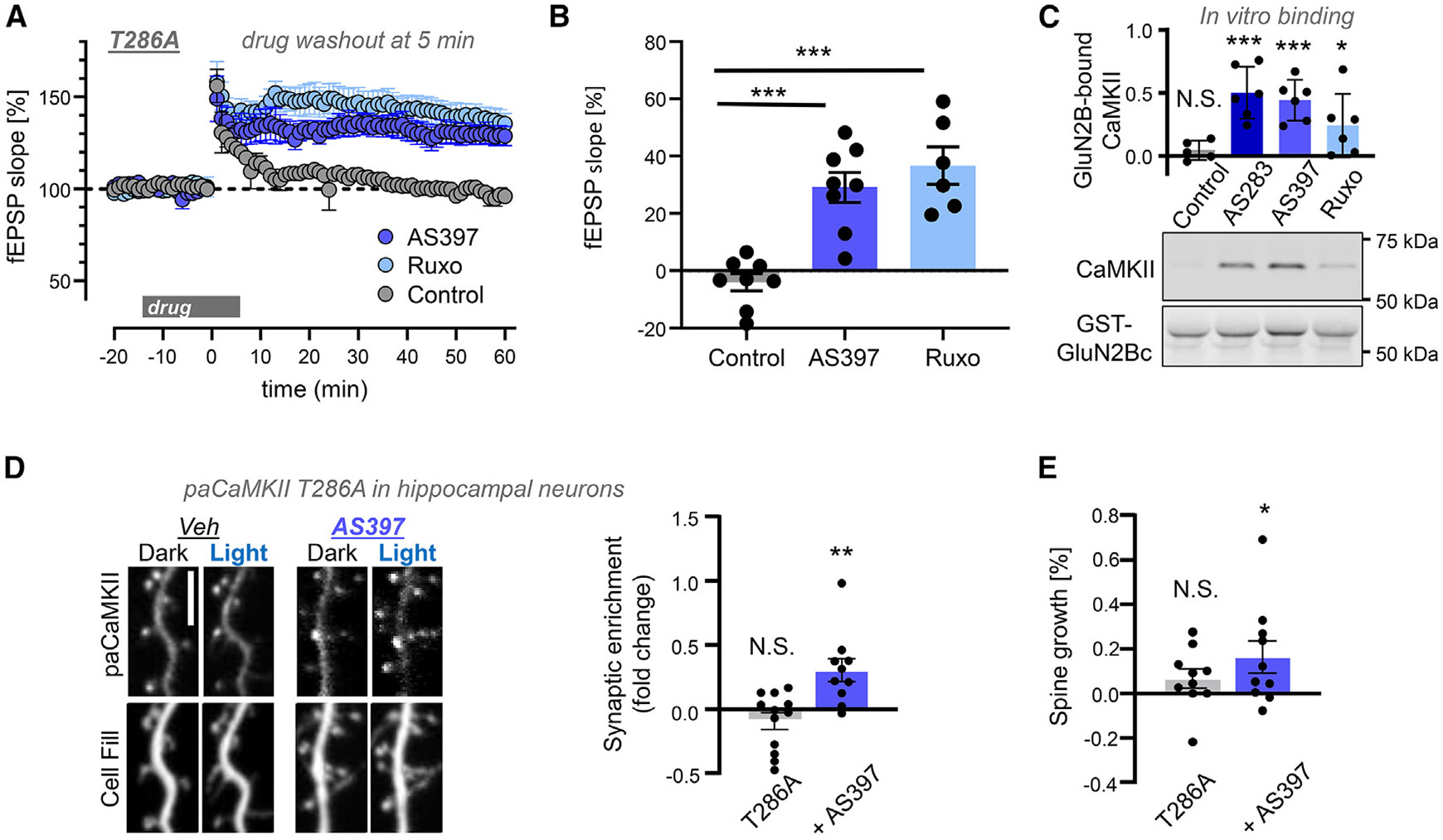
ATP-competitive CaMKII inhibitors enable LTP without pT286 Data show mean ± SEM. (A) LTP was assessed by the field excitatory postsynaptic potential (fEPSP) slope at the CA3 to CA1 synapse in hippocampal slices of CaMKII T286A mutant mice. LTP was stimulated by 2× HFS(2 × 1 s 100 Hz with 10 s interval) 20 min after recording of fEPSP baseline; 10 μM drug was added 15 min prior to the stimulation. The ATP-competitive inhibitors AS397 and ruxolitinib(Ruxo) rescued LTP in T286A mice when they were washed out at 5 min after HFS, as shown in a timeline of fEPSP recordings (*n* = 8, 8, and 6 slices for control, AS397, and Ruxo, respectively). (B) The rescue is demonstrated by the change in fEPSP slope during the last 5 min or recording (***p < 0.001 by Bonferroni’s multiple comparison test following one-way ANOVA). (C) Representative immunoblots of *in vitro* binding reaction of purified CaMKII to GST-GluN2Bc that was immobilized on microtiter plates, with quantification in arbitrary relative units. In this case, Ca^2+^/CaM in the absence of nucleotide or inhibitors (control) induced no significant binding, but significant binding was detected in the presence of 10 μM AS283, AS397, or Ruxo (one-sample t test; ***p < 0.001 and *p < 0.05). (D) Representative images and quantification of dendritic spine localization of the photoactivatable GFP-paCaMKII T286A mutant in cultured hippocampal neurons. The addition of 10 μM AS397 enabled synaptic enrichment in response to light stimulation (*n* = 12, 10 cells; one-sample t test; T286A *p* = 0.1773; T286A + AS397 *p* = 0.0081; **p < 0.01). Scale bar: 5 μm. (E) Photoactivation of paCaMKIIα T286A in hippocampal neuron caused a significant increase in spine area only in the presence of 10 μM AS397 application *(*p* < 0.05 in one sample t test; *n* = 10 and 12 for T286A alone and for T286A + AS397, respectively).

Our recent study indicated that the requirement of CaMKII pT286 for LTP is not for the generation of autonomous CaMKII activity but for the regulation of CaMKII binding to GluN2B.^6^ One of the most convincing lines of evidence for this was provided by the fact that induction of hippocampal CA1 LTP by high-frequency stimulation (HFS; 2× 100 Hz with 10 s interval) was enabled even in slices from T286A mice when we added the ATP-competitive CaMKII inhibitor AS283. In contrast to other CaMKII inhibitors, ATP-competitive CaMKII inhibitors do not interfere with CaMKII binding to GluN2B.^9,23^ AS283 even directly enhanced GluN2B binding and thereby circumvented the requirement for pT286.^6^ Here, we show such rescue of LTP induction in T286A also by two other ATP-competitive CaMKII inhibitors: AS397^24^ and ruxolitinib^24,25^ (Figures 1A and 1B). Like AS283, either of these inhibitors directly enhanced CaMKII binding to GluN2B *in vitro* (Figures 1C and S1A) and allowed CaMKII binding to GluN2B in HEK cells (Figures S1B-S1D). In hippocampal neurons, AS397 also enabled light-induced movement of a photoactivatable CaMKII T286A mutant to excitatory spine synapses and spine growth (Figures 1D and 1E), as expected. AS397 was developed alongside AS283 as a CaMKII-selective inhibitor, whereas ruxolitinib is better known as a JAK/STAT inhibitor.^24,25^ Notably, despite some similarities, both AS397 and ruxolitinib are structurally distinct from AS283 (Figure S1E).

### Post-induction requirement for CaMKII activity but not pT286 in LTP

**Figure 2.**
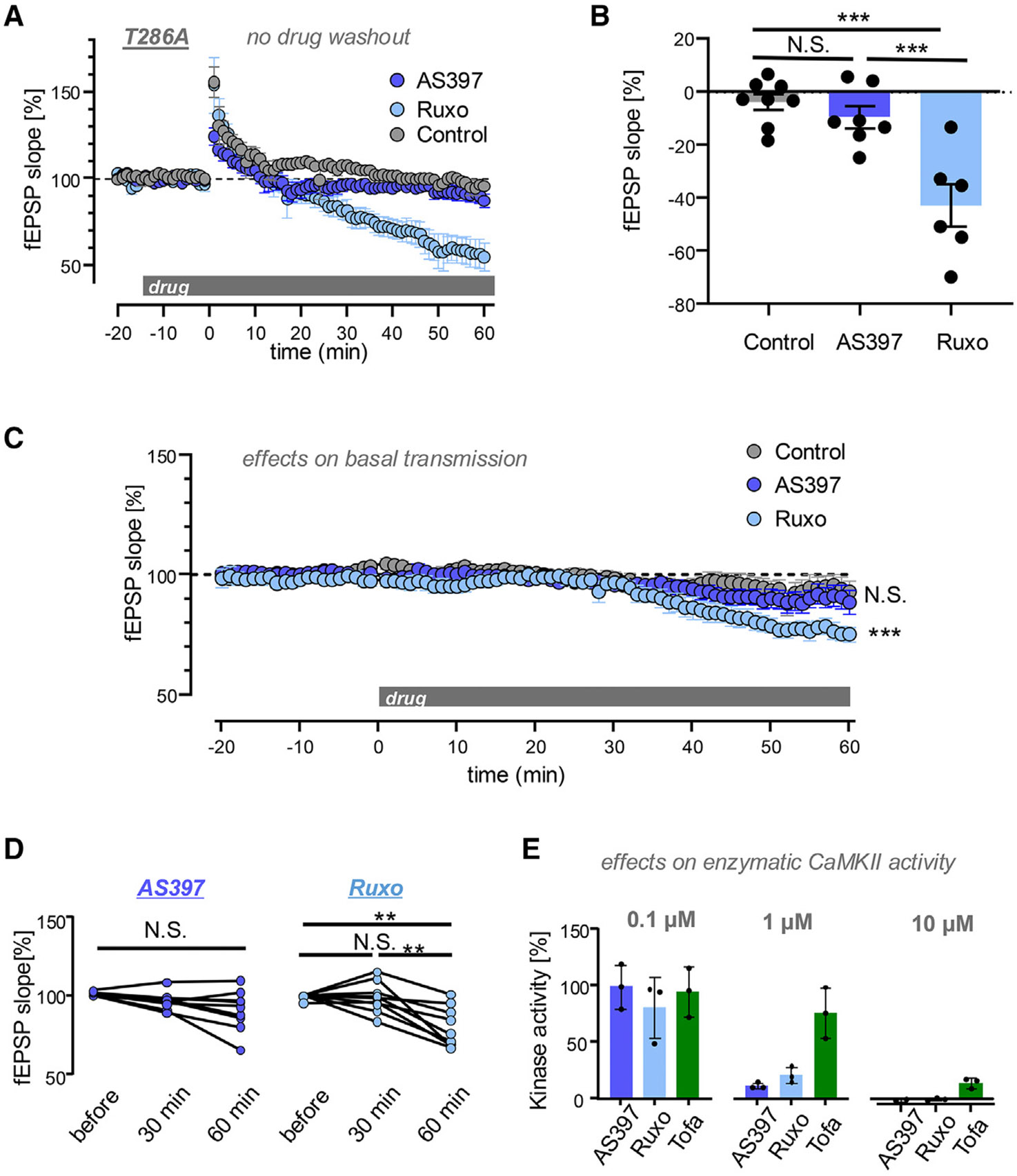
The ATP-competitive CaMKII inhibitor AS397 affects LTP but not basal synaptic transmission Strength of synaptic transmission was assessed by fEPSP slope at the CA3 to CA synapses in hippocampal slices in the presence of various drugs (10 μM). Data show mean ± SEM. (A) When the ATP-competitive inhibitors were not washed out after HFS, no rescue of LTP was seen in the T286A mice, as shown in a timeline of fEPSP recordings (*n* = 8, 8, and 6 slices for control, AS397, and ruxolitinib [Ruxo], respectively). (B) During the last 5 min of recording, AS397 reduced the fEPSP back to baseline, whereas Ruxo caused a further decrease below baseline (***p < 0.001 by Bonferroni’s multiple comparison test following one-way ANOVA). (C) An apparent mild decrease in basal synaptic transmission was observed in wild-type slices for Ruxo, AS397, and control without drug (*n* = 9 slices for each condition), but significance compared to control was reached only for Ruxo (***p < 0.001) (D) Compared to baseline before drug addition, only Ruxo, but not AS397, significantly reduced fEPSP slopes and only at 60 min, not at 30 min, after drug addition (**p < 0.01 repeated measured one-way ANOVA with Bonferroni’s post hoc analysis). (E) Biochemical assays *in vitro* showed that enzymatic CaMKII activity was dramatically reduced by 1 μM AS397 or Ruxo. At 10 μM, AS397 and Ruxo completely eliminated any measurable CaMKII activity, but tofacitinib (Tofa) also started to cause significant inhibition.

For the rescue of LTP induction in T286A mice by AS397 or ruxolitinib (and previously by AS283),^6^ the inhibitors were washed out 5 min after the LTP stimuli (see Figures 1A and 1B). Thus, the continued maintenance of LTP that was observed under these conditions did not depend on the pT286-induced autonomous CaMKII activity, as pT286 is prevented by the T286A mutation. However, some form of CaMKII activity appeared to be required because LTP was reversed when AS397 or ruxolitinib was not washed out and instead allowed to remain on the slices for the remainder of the recordings (Figures 2A and 2B). Thus, a post-induction phase of LTP requires enzymatic CaMKII activity, even if LTP induction does not.

### Distinct effects of different inhibitors on basal synaptic strength

Notably, whereas AS397 reversed the LTP in T286A mice back to baseline, ruxolitinib reduced synaptic strength even below baseline (Figures 2A and 2B). Thus, we next compared the effects of the two inhibitors on baseline synaptic strength without LTP induction in wild-type slices. At 30 min after drug addition, neither AS397 nor ruxolitinib showed any effect on baseline transmission (Figures 2C and 2D). However, at 60 min, ruxolitinib reduced baseline transmission mildly but significantly (Figures 2C and 2D). By contrast, the slight apparent decrease observed with AS397 at 60 min was not statistically significant compared to baseline or the slight apparent decrease seen also in the control condition without the drug (Figures 2C, 2D, and S2A).

In order to further clarify potential effects on baseline transmission, we next compared AS283 (another CaMKII-selective inhibitor)^6^ and tofacitinib (another clinically used JAK/STAT inhibitor that is not reported to inhibit CaMKII, although at high concentrations, we found a significant reduction of CaMKII activity also with tofacitinib; Figure 2E).^24,25^ The slight apparent decrease for either of these inhibitors was not statistically significantly different from the slight apparent decrease in the control without the drug (Figures S2B and S2C). However, in contrast to control and AS397, the slight decrease compared to baseline before drug administration was statistically significant (Figure S2D).

Overall, the significant but mild decrease in basal transmission seen with ruxolitinib may not be fully sufficient to explain the stronger LTP reversal below baseline; however, neither effect is due to CaMKII inhibition: The more selective inhibitor AS397 reversed LTP only to baseline and had no significant effect on basal transmission, indicating that ATP-competitive CaMKII inhibitors can cause specific reversal of LTP.

### Reversal of LTP by ATP- but not CaM-competitive CaMKII inhibitors

**Figure 3.**
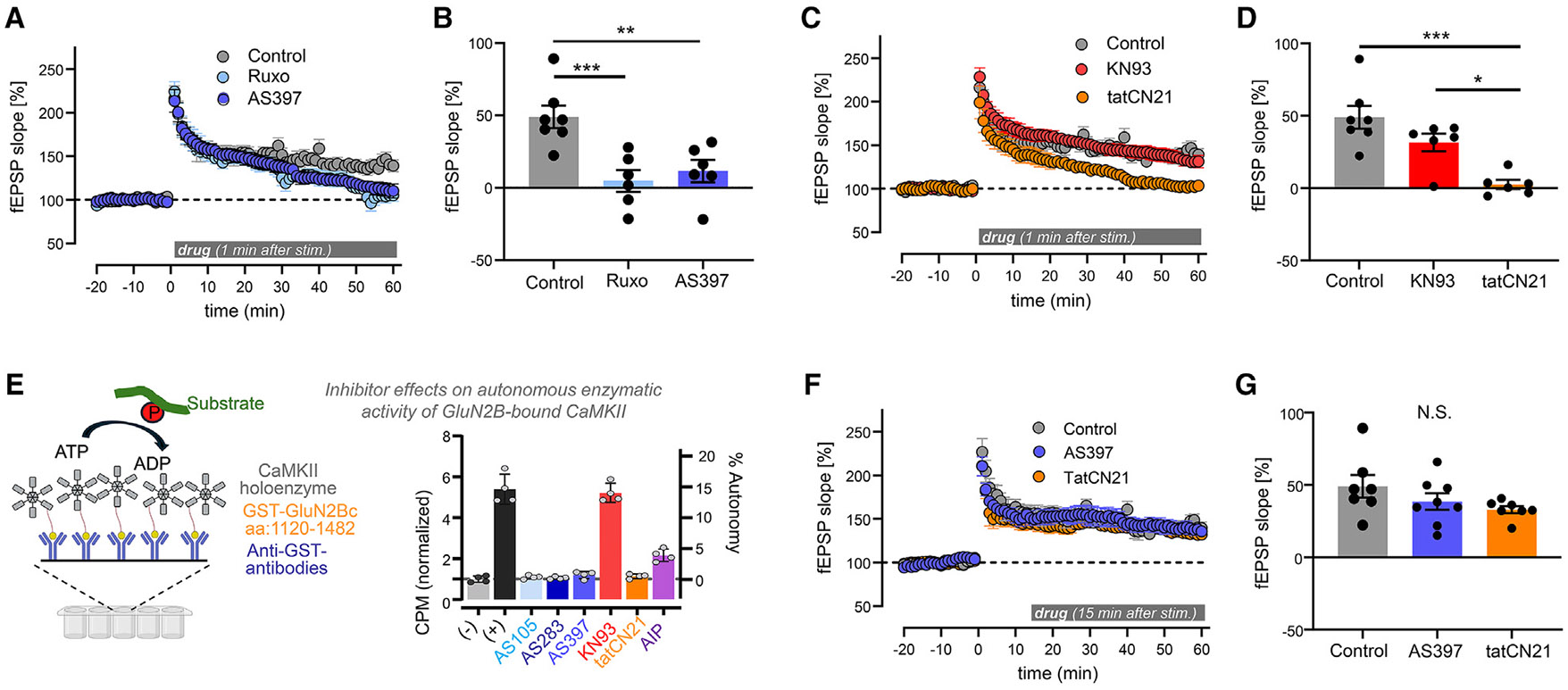
Differential effect of different classes of CaMKII inhibitors on LTP maintenance in wild-type mice LTP was induced by 2× HFS and measured by the fEPSP slopes at the CA3 to CA synapses in hippocampal slices from wild-type (WT) mice. Drug (10 μM unless noted otherwise) was added as indicated. Data show mean ± SEM. (A) When added 1 min after LTP induction, the ATP-competitive inhibitors AS397 and ruxolitinib (Ruxo) both reduced fEPSP slopes back to baseline within 60 min (*n* = 7, 6, and 6 slices for control without drug, Ruxo, and AS397, respectively). (B) Significant reduction of LTP during the last 5 min of recording was observed for both AS397 and Ruxo (**p < 0.005 and ***p < 0.001 by Bonferroni multiple comparison test following one-way ANOVA). (C) When added 1 min after LTP induction, the peptide inhibitor tatCN21 (5 μM), but not the CaM-competitive inhibitor KN93, reduced fEPSP slopes back to baseline within 60 min (*n* = 6 for KN93 and tatCN21). (D) During the last 5 min of recordings, only tatCN21, but not KN93, caused a significant reduction of fEPSP slope compared to the no-drug control, as also shown in the other images, and tatCN21 also caused significant reduction compared to KN93 (***p < 0.001 and *p < 0.05 by one-way ANOVA followed by Bonferroni multiple comparison test). (E) The effect of different inhibitors (10 μM) on the autonomous activity of GluN2B-bound CaMKII was tested *in vitro* as illustrated in the schematic on the right. CaMKII binding was induced by Ca^2+^/CaM, which was then washed out before measuring the Ca^2+^/CaM-independent autonomous activity of the bound kinase. Negative control (−) contained no kinase and positive control (+) no inhibitor. All ATP-competitive inhibitors (AS105, AS283, and AS397) and tatCN21 completely blocked activity. KN93 had no effect on activity, and AIP reduced activity but did not block it completely. (F) When added 15 min after LTP induction, the ATP-competitive inhibitor AS397 had no apparent effect on LTP maintenance (*n* = 8 slices). (G) During the last 5 min of recording, addition of ATP at 15 min after LTP induction had no significant effect on fEPSP slope compared to the control without the drug, which is also shown in the other images.

Next, we tested the effects of CaMKII inhibitors on LTP reversal in wild-type mice and found effective LTP reversal by both AS397 and ruxolitinib when added 1 min after LTP induction (Figures 3A and 3B). In this case, neither drug appeared to cause a decrease below baseline. However, ruxolitinib is expected to show such a decrease upon further extended incubation times, as was indeed observed for ruxolitinib, but not AS397, after LTP in T286A mice (see Figures 2A and 2B) or for basal transmission in wild-type mice (see Figures 2C and 2D). In contrast to the ATP- competitive inhibitors, the CaM-competitive CaMKII inhibitor KN93 did not reverse LTP (Figures 3C and 3D), indicating that the enzymatic CaMKII activity that is required after LTP induction is a form of Ca^2+^-independent autonomous activity. This suggested autonomous activity induced by GluN2B binding, as pT286-induced autonomous activity was ruled out in our earlier experiments (shown in Figures 1A and 1B) and autonomous CaMKII activity induced by S-nitrosylation or oxidation is not required for HFS-induced LTP.^26^

Notably, the peptide inhibitor tatCN21 (which binds to the CaMKII T-site) did reverse LTP when added 1 min after induction (Figures 3C and 3D). This was initially surprising, as tatCN21 was assumed to not inhibit the activity of GluN2B-bound CaMKII. However, biochemical kinase activity assays with purified protein showed that the autonomous CaMKII activity that is induced by *in vitro* binding to the immobilized GluN2B C-tail is blocked not only by the ATP-competitive inhibitors AS105, AS283, and AS397 but also by tatCN21 (Figure 3E). This may be explained by additional binding of tatCN21 to the substrate binding S-site of CaMKII, as indeed indicated by a crystal structure of a CaMKII kinase domain bound to the CaMKIIN peptide that includes the CN21 sequence.^27^ Thus, while tatCN21 prevents CaMKII from binding to GluN2B,^15^ it can still inhibit the activity of GluN2B-bound CaMKII even without disrupting this binding. A different peptide inhibitor of CaMKII, autocamtide-2-related inhibitory peptide (AIP), also inhibited the autonomous activity of GluN2B-bound CaMKII; however, in contrast to tatCN21, it did not block this activity completely. The CaM-competitive KN93 had no effect, as expected (Figure 3E).

### Autonomous CaMKII activity is required in an early post-induction phase of LTP

While we here observed LTP reversal by applying 5 mM tatCN21 1 min after LTP induction (Figures 3C and 3D), we previously did not see reversal with application at 15 min after LTP induction, at least in hippocampal slices from rat.^16^ Here, we first confirmed that such a later application of 5 μM tatCN21 also did not reverse LTP in slices from mouse (Figures 3F and 3G). Most importantly, at 15 min, AS397 also failed to reverse LTP (Figures 3F and 3G). This is consistent with both drugs targeting the same pool of CaMKII and demonstrates that enzymatic CaMKII activity is required only during an early post-induction phase of LTP. As will be discussed, the early post-induction requirement for enzymatic CaMKII activity provides an objective definition for the phase of LTP expression.

### LTP expression requires CaMKII activity of GluN2B-bound CaMKII

**Figure 4.**
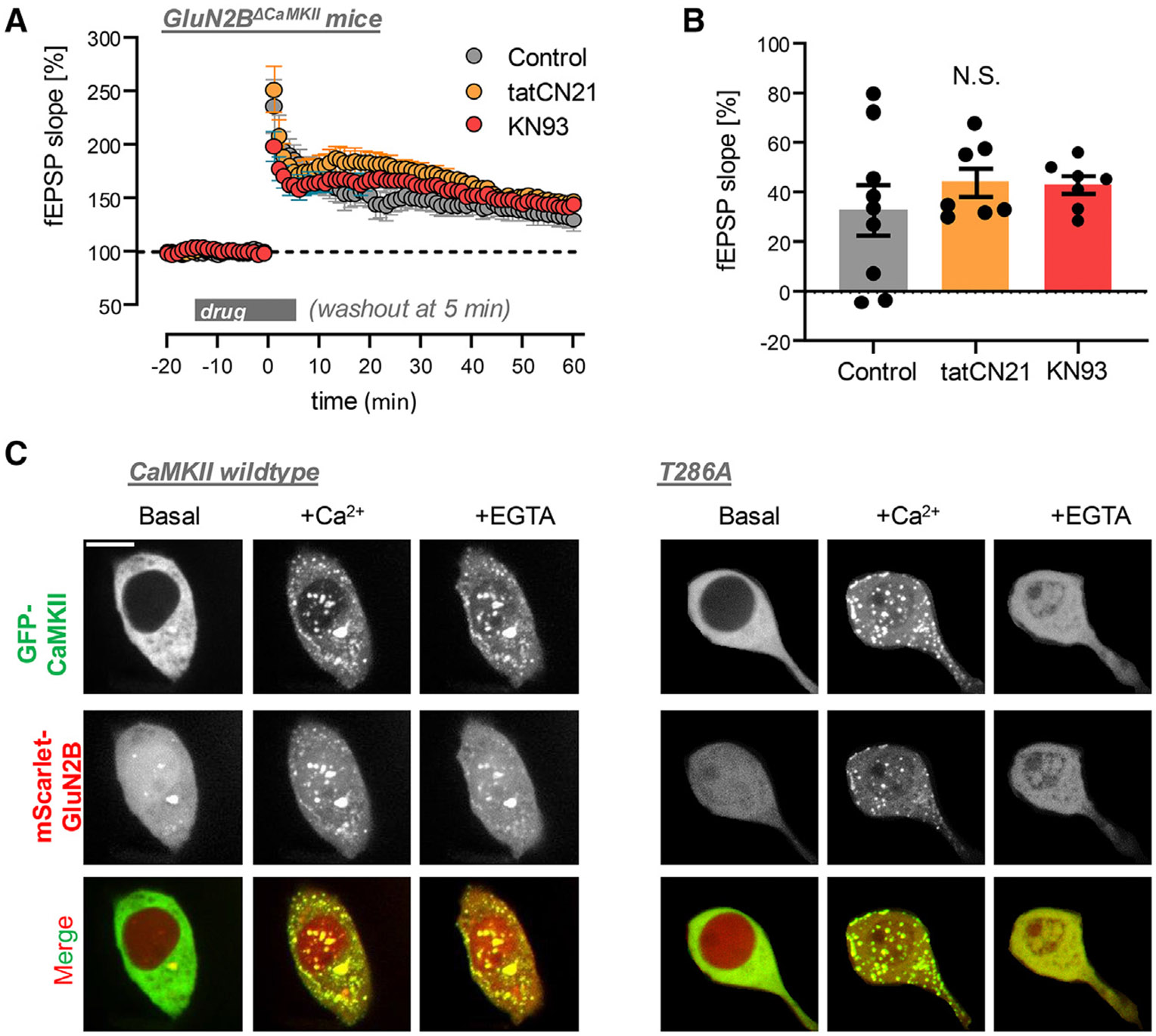
Probing the role of CaMKII/GluN2B binding versus co-condensation in LTP Data show mean ± SEM. (A) In slices from mice with the GluN2B^ΔCaMKII^ mutant that prevents CaMKII binding to GluN2B, neither 5 μM tatCN21 nor 10 μM KN93 (two CaMKII inhibitors that block LTP induction in wild-type mice) reduced LTP compared to control without drug (*n* = 9,8, and 7 slices for control, tatCN21, and KN93, respectively), showing that the LTP seen in the mutant mice was enabled by compensatory mechanisms and different from LTP in wild type. Drug was added 15 min before and washed out 5 min after LTP induction, as indicated. LTP was induced by 2× HFS and measured by the fEPSP slopes at the CA3 to CA synapses. (B) During the last 5 min of recording, no statistical difference was detected in the fEPSP slopes in the tatCN21, KN93, or control condition (one-way ANOVA). (C) Co-condensation of CaMKII wild type with the cytoplasmic C-tail of GluN2B in HEK cells is triggered by a Ca^2+^ stimulus with ionomycin and then maintained after chelating Ca^2+^ with EGTA (even if at a reduced level). For a CaMKII T286A mutant, Ca^2+^ triggers at least as much co-condensation, but these condensates are then fully reversed after chelating Ca^2+^ with EGTA. Scale bar: 10 μm.

If LTP induction requires CaMKII binding to GluN2B and LTP expression requires autonomous activity of the GluN2B-bound CaMKII, why is there still LTP in mice with GluN2B mutations that prevent this binding? In one of these strains (GluN2B^ΔCaMKII^, with the L1298A/R1300N mutations), LTP is reported to be reduced by half^28^; in the other strain (which carries an additional S1303D mutation), no LTP reduction was observed at all.^29^ It has been argued that this may be due to compensatory effects in these constitutive mouse mutants.^6^ Even though this argument is supported by the fact that more acute disruption of CaMKII binding to GluN2B had much more severe effects on LTP,^19,30^ it may appear somewhat handwaving. However, such compensatory effects can be tested directly: if the remaining LTP in these mice is indeed due to compensation, then it should no longer be blocked by drugs that prevent CaMKII binding to GluN2B (and thus block LTP in wild-type mice). Thus, we decided to test the effects of tatCN21 and KN93 on LTP induction in the GluN2B^ΔCaMKII^’ mice (Figures 4A and 4B). Notably, even though we showed here that tatCN21 can inhibit the activity of GluN2B-bound CaMKII, we have shown previously that it can prevent the induction of this binding.^15^ Similarly, even though KN93 does not affect the autonomous activity of GluN2B-bound CaMKII once the binding is established, it prevents the stimulation by Ca^2+^/CaM that is required for both activation and induction of the GluN2B binding.^12,13^ Indeed, we found that LTP induction in the GluN2B^△CaMKII^ mice is not reduced at all by tatCN21 or KN93 (Figures 4A and 4B), providing direct evidence that LTP in these mice is enabled by compensatory mechanisms that are absent in wild-type mice. Notably, in our hands, the LTP in GluN2B^DCaMKII^ mice in the absence of the drug was only slightly lower compared to wild-type mice; however, a direct quantitative comparison cannot be made, as the wild-type mice tested here were not littermates of the mutants.

### Mixed contribution of CaMKII activity to the compensatory effects in GluN2B mutant mice

Even though the mechanism of the compensatory effects that we have demonstrated for the GluN2B^ΔCaMKII^ mice is not of physiological importance, we decided to test whether or not it involves the enzymatic activity of non-GluN2B-bound CaMKII. To this end, we tested the effect of two inhibitors that completely reversed LTP in wild-type mice (see Figure 3) on LTP reversal in the GluN2B^ΔCaMKII^ mice. The addition of either AS397 or tatCN21 at 1 min after LTP induction appeared to reduce LTP also in these mice; however, this apparent reduction in LTP was not statistically significant compared to the no-drug control (Figures S3A and S3B). Additionally, while the potentiation appeared to be reduced after drug incubation, a statistically significant potentiation compared to baseline remained in the slices from the GluN2B^ΔCaMKII^ mice (Figure S3C), whereas no potentiation remained in the wild-type slices (see Figure 3). These results suggest that the compensatory effects in the GluN2B^ΔCaMKII^ rely partially, but not entirely, on the enzymatic activity of CaMKII that is not bound to GluN2B.

### Regulation of CaMKII co-condensation with GluN2B does not support LTP maintenance

Ca^2+^/CaM stimulation induces both binding and co-condensation of CaMKII and GluN2B.^20,21^ However, maintenance of the co-condensation beyond the Ca^2+^ stimulus requires pT286,^20^ at least *in vitro*. By contrast, pT286 is required for neither the maintenance of traditional high-affinity GluN2B binding^13^ nor for the maintenance of LTP (see Figures 2A and 2B), indicating that LTP maintenance could be mediated by the traditional binding but not by CaMKII co-condensation with GluN2B. However, as our knowledge of the regulation of co-condensation is currently entirely based on cell-free biochemical studies, we decided to test this regulation within cells. This was achieved by co-expressing the GFP-CaMKII wild type or T286A mutant with a soluble mScarlet-labeled non-membrane targeted fragment of the cytoplasmic C-tail of GluN2B in HEK cells. Similarly as predicted from the biochemical studies, co-condensation of CaMKII with GluN2B was triggered by inducing Ca^2+^ signals with ionomycin (Figures 4C and S4A). This co-condensation also allowed CaMKII to enter the nucleus, a compartment from which it is normally largely excluded due to its size. As expected from co-condensation, the CaMKII and GluN2B clusters overlapped (Figure 4C). When expressed alone, CaMKII did not form any such clusters by itself after the same treatment (Figure S4B); GluN2B alone showed only milder clustering, even without stimulation (Figures 4C and S4B). Upon subsequent chelation of Ca^2+^ with EGTA, significant co-condensation of CaMKII and GluN2B was retained for the CaMKII wild type but completely reversed for the CaMKII T286A mutant (Figure 4C). In contrast to restoration of LTP in the T286A mutant (see Figures 1A and 1B), the ATP-competitive inhibitors AS397 and ruxolitinib did not restore maintenance of co-condensation for the T286A mutant (Figure S4C). Thus, the differential requirement of pT286 indicates that cellular co-condensation of CaMKII with GluN2B does not mediate LTP maintenance.

## DISCUSSION

**Figure 5.**
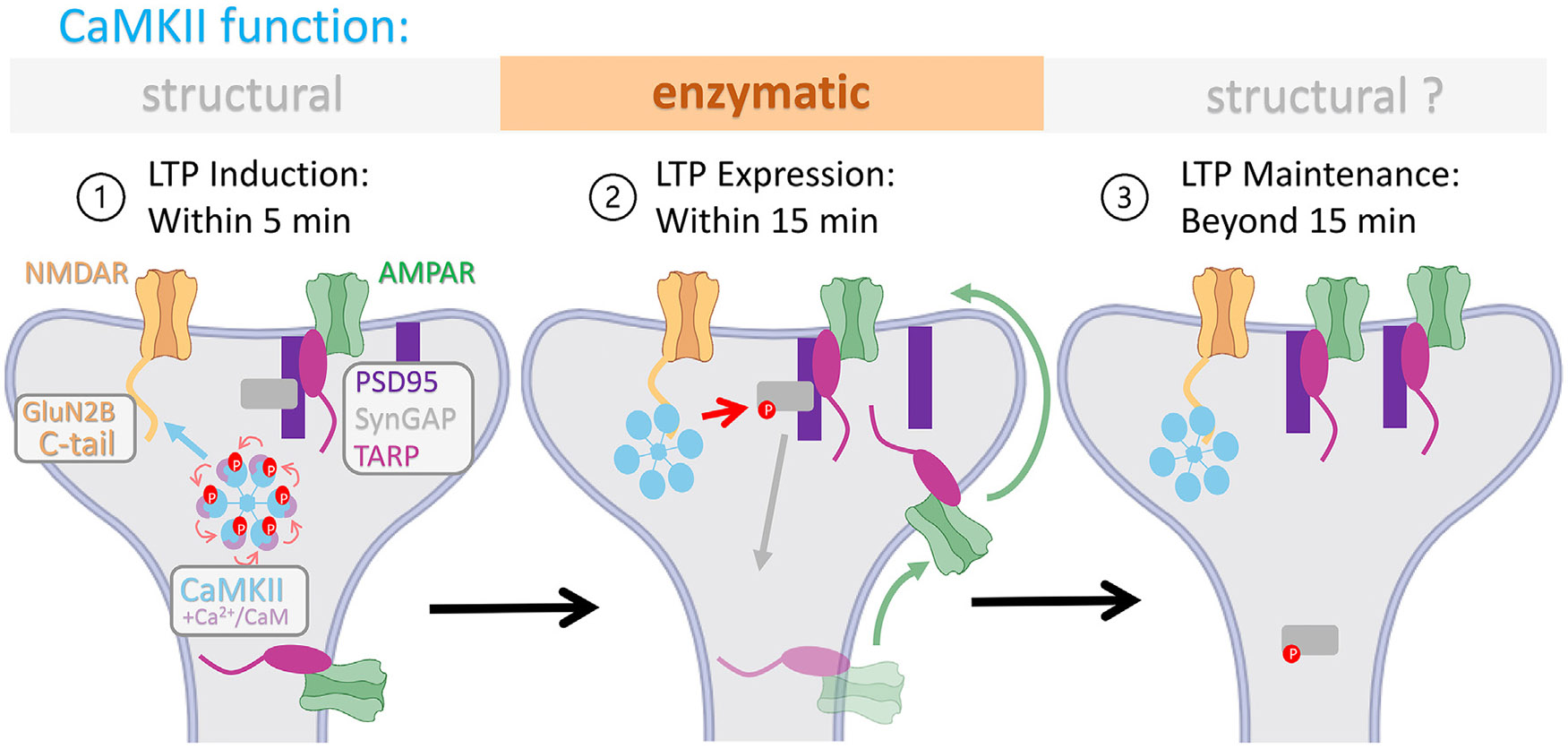
Illustration of CaMKII structural and enzymatic functions in the phases of LTP (1) Enzymatic CaMKII activity is not required for LTP induction within the first 5 min of stimulation. The structural CaMKII function that is required instead is regulated binding to GluN2B, which primes CaMKII for localized Ca^2+^-independent autonomous activity. The CaMKII autophosphorylation at T286 (indicted by arrows within the holoenzyme) aids in this binding step but is not required subsequently. (2) The autonomous enzymatic activity of GluN2B-bound CaMKII mediates LTP expression within the first 15 min. During LTP expression, CaMKII phosphorylates SynGAP to remove it from the synapse to allow accumulation of TARPs and, thereby, AMPARs. Additionally, direct phosphorylation of TARPs may further promote AMPAR trapping during LTP expression. (3) Beyond 15 min, the structural functions of GluN2B-bound CaMKII may continue to promote LTP maintenance, but the enzymatic activity is no longer required.

This study fundamentally elucidates the contribution of structural versus enzymatic CaMKII functions to the phases of LTP that are induced by HFS at the hippocampal CA3 to CA1 synapse; the resulting model is described here and illustrated in Figure 5. We show that LTP induction requires CaMKII binding to GluN2B. This binding directly generates Ca^2+^-independent autonomous CaMKII activity,^13^ but this enzymatic activity is not required within the first 5 min of LTP induction. However, the second phase, LTP expression, does require enzymatic activity, specifically the autonomous activity of the GluN2B-bound CaMKII that was generated during the induction phase. The likely substrates and mechanisms are discussed below, but neither Ca^2+^/CaM-stimulated activity nor autonomous activity generated by pT286 is required at this point. The third phase, LTP maintenance, begins around 15 min after induction and no longer requires any enzymatic CaMKII activity, neither stimulated nor autonomous. This LTP maintenance may still require structural functions of the CaMKII binding to GluN2B^19,30,31^ but is not mediated by their more recently described co-condensation.

Two apparent sidenotes of these findings are worth high-lighting: the involvement of pT286 and condensation. For almost 40 years, CaMKII pT286 and the resulting autonomous activity have been proposed to mediate long-term memory, and this model is still widely propagated even though it is outdated. Our findings show that pT286 is indeed crucial for LTP but during the induction phase to promote GluN2B binding rather than during expression or maintenance. This is clearly demonstrated by our experiments in T286A mice: both LTP expression and maintenance were enabled despite the T286A mutation when the requirement for pT286 in LTP induction was circumvented by directly enhancing GluN2B binding with AS397 or ruxolitinib (with drug washout at 5 min after LTP induction). We have recently reported similar findings with another ATP-competitive CaMKII inhibitor, AS283, but with a much more limited number of repetitions.^6^ Notably, this requirement for pT286 in LTP induction but not maintenance helps explain earlier behavioral studies: the T286A mice are severely impaired in learning, but when they learn after more extensive training, their memory is normal.^32^ It will be interesting to see if the learning impairments of T286A mice can also be restored by ATP-competitive CaMKII inhibitors.

For CaMKII co-condensation with GluN2B, our findings in HEK cells confirm prior results with cell-free assays^20,21^ indicating that Ca^2+^/CaM stimulation is sufficient for the induction of condensation but that pT286 is required for its maintenance beyond the initial stimulus. This differential requirement for pT286 clearly indicates that CaMKII co-condensation with GluN2B is not suitable to mediate the maintenance phase of LTP. However, this should not be misconstrued to mean that condensation in general (or the co-condensation of CaMKII with GluN2B in particular) does not have important functions in the organization of the synapse.^22^

One previous study appears to be in conflict with our findings regarding enzymatic CaMKII activity in LTP expression: results with a photoactivatable CaMKII inhibitor indicated that there is no requirement for enzymatic CaMKII activity at all after 2 min of LTP induction.^33^ However, the photoactivatable inhibitor used a derivate of the AIP peptide, and we have shown here that AIP does not completely block the enzymatic activity of GluN2B-bound CaMKII, which is the specific CaMKII activity implicated by our study. Additionally, access to GluN2B-bound CaMKII could be further hindered by the fusion of the AIP peptide with the light-oxygen-voltage-sensing (LOV) domain that mediates the photoactivation. Notably, before our study, it was assumed that peptide inhibitors of CaMKII would not inhibit GluN2B-bound CaMKII at all.^9^ Thus, even the partial inhibition by AIP (and certainly the full inhibition by tatCN21) came as a surprise.

Our model directly implies that the enzymatic CaMKII activity that is required for LTP is highly localized at the postsynaptic site, which helps explain how the input specificity of LTP (i.e., the fact that LTP is induced only at the stimulated synapse and not at its neighbors) is generated by the input-specific accumulation of CaMKII.^34^ Such local function is also consistent with a role for CaMKII-mediated synaptic trapping of AMPARs during LTP.^35^ But what are the specific CaMKII substrates that are involved? While CaMKII-mediated phosphorylation of GluA1 at S831 would contribute to increased single-channel conductance of AMPARs,^36,37^ this phosphorylation is not required for LTP and not essential for synaptic AMPAR insertion or trapping. Phosphorylation of the AMPAR auxiliary subunit TARP-γ8 at S277 and S281 remains a potential option, as this clearly promotes synaptic strength.^38,39^ However, it remains unclear if this regulates basal strength versus LTP, if it is required acutely during LTP, and if it is mediated by CaMKII or by PKC.^6,9,38-40^ Another compelling possibility was just published this year^41^: phosphorylation of SynGAP at S1108 and S1138 induces its removal from synapses. In turn, this enables synaptic accumulation of TARP-γ8 and, thereby, AMPARs.^41^ While the responsible kinase is not entirely clear in this case either,^9,40^ its phosphorylation by CaMKII has been suggested previously.^42^

Independent of the specific substrate(s) involved, our findings provide a new objective definition and separation of the early phases of LTP: the induction phase of LTP includes the signal processing that leads to CaMKII binding to GluN2B, which directly primes CaMKII for subsequent Ca^2+^/CaM-independent autonomous activity. Then, the expression phase of LTP is mediated by the local autonomous activity of the GluN2B-bound CaMKII and involves phosphorylation of substrates that lead to increased synaptic AMPAR numbers. This concept is also consistent with the observation that CaMKII binding to GluN2B increases the motility of NMDARs within the postsynaptic den-sity (PSD)^43^: the motility of the CaMKII/NMDAR complex that forms during LTP induction would allow access to all substrates within the PSD. At the same time, the necessity of this large complex to move within the PSD would help explain the surprisingly long time window of the LTP expression phase indicated here.

Overall, the current study provides a fresh example that CaMKII regulation of synaptic plasticity remains full of surprises despite four decades of intensive research. We hope that our results will help reshape the focus on the most fruitful areas by moving beyond the long-standing but outdated hypothesis about a potential role of pT286 in LTP maintenance and encouraging more detailed research on the relevant enzymatic and structural functions of CaMKII that are required instead.

### Limitations of the study

While our study clarifies the specific CaM KII functions required for LTP induction and expression, the CaMKII functions in LTP maintenance remain unclear, other than showing that neither pT286 nor enzymatic kinase activity is required. Specifically, we show that LTP induction and expression both require CaMKII binding to GluN2B, but the previously suggested role in LTP maintenance needs further investigation. Also, while our study suggests that co-condensation of CaMKII with GluN2B is not required for LTP maintenance, further studies on this aspect will still be illuminating. Additionally, for the other phases of LTP, a potential role of this co-condensation remains entirely unclear.

## RESOURCE AVAILABILITY

### Lead contact

Further information and requests for resources and reagents should be directed to and will be fulfilled by the lead contact, K. Ulrich Bayer (ulli.bayer@ucdenver.edu).

### Materials availability

Plasmids used in this work will be available upon request.

### Data and code availability

- The datasets generated during this study are available at Mendeley Data: http://www.doi.org/10.17632/d4r2vx4n4t.1.
- Code: not applicable.
- Any additional information required to reanalyze the data reported in this paper is available from the lead contact upon request.

## STAR★METHODS

### EXPERIMENTAL MODEL AND SUBJECT DETAILS

All animal procedures were approved by the University of Colorado Institutional Animal Care and Use Committee (IACUC) and carried out in accordance with National Institutes of Health best practices for animal use. The University of Colorado Anschutz Medical Campus is accredited by the Association for Assessment and Accreditation of Laboratory Animal Care, International (AAALAC). All animals were housed in ventilated cages on a 12 h light/12 h dark cycle and were provided *ad libitum* access to food and water. Male wildtype, T286A mutant, and GluN2B^ΔCaMKII^ mutant mice (on a C57BL/6 background) from heterozygous breeder pairs (8–12 weeks old) were used for slice electrophysiology. Mixed sex pups from Sprague-Dawley rats (P0, Charles River) were used to prepare dissociated hippocampal cultures for imaging. The mutant mice used here were described previously: the GluN2B^ΔCaMKII^ line was kindly provided by Johannes Hell^28,44^; and the T286A line was kindly provided by Ryohei Yasuda with kind permission from Karl Peter Giese.^8^

### METHOD DETAILS

#### Material and DNA constructs

Material was obtained from Sigma, unless noted otherwise. CMV-mEGFP(A206K)-paCaMKII was a gift from Hideji Murakoshi (Addgene plasmid # 165438). Like this paCaMKII construct, all constructs used here that contain GFP actually encode for the EGFP with additional A206K mutation to minimize dimerization.

#### Protein purification

Expression and purification of CaMKIIα, CaM, GST-GluN2Bc and GST-GluA1 were conducted according to established protocol described in detail previously.^13,45-47^ CaMKIIα was purified from a baculovirus/Sf9 cell expression system. CaM and GST-GluN2Bc wild type and mutant constructs were purified from BL21 bacteria.

#### Hippocampal slice preparation

Preparation of and recording from hippocampal slices was performed as described previously.^6^ wild type and mutant mouse hippocampal slices were prepared using P56-70 mice. Isoflurane anesthetized mice were rapidly decapitated, and the brain was dissected in ice-cold high sucrose solution containing 220 mM sucrose, 12 mM MgSO_4_,10 mM glucose, 0.2 mM CaCl_2_, 0.5 mM KCl, 0.65 mM NaH_2_PO_4_, 13 mM NaHCO_3_, and 1.8 mM ascorbate. Transverse hippocampal slices (400 μm) were made using a tissue chopper (McIl-wain) and transferred into 32°C artificial cerebral spinal fluid (ACSF) containing 124 mM NaCI, 3.5 mM KCl, 1.3 mM NaH_2_PO_4_, 26 mM NaHCO_3_, 10 mM glucose, 2 mM CaCl_2_,1 mM MgSO_4_, and 1.8 mM ascorbate. All slices were recovered in 95% O_2_/5% CO_2_ for at least 1.5 h before experimentation.

#### Extracellular field recordings

For electrophysiological slice recording experiments, a glass micropipette (typical resistance 0.4 to 0.8 MΩ when filled with ACSF) was used to record field excitatory postsynaptic potentials (fEPSPs) from the CA1 dendritic layer in response to stimulation of the Schaffer collaterals using a tungsten bipolar electrode. Slices were continually perfused with 30.5° ± 0.5°C ACSF at a rate of 2.5 ± 0.5 mL/min during recordings. Stimuli were delivered every 20 s, and three responses (1 min) were averaged for analysis. Data were analyzed using WinLTP software with slope calculated as the initial rise from 10 to 60% of response peak. Input/output (I/O) curves were generated by increasing the stimulus intensity at a constant interval until a maximum response or population spike was noted to determine stimulation that elicits 50% of maximum slope. Paired-pulse recordings (50-ms interpulse interval) were acquired from 50% max slope, and no differences in presynaptic facilitation were seen in mutant slices. A stable baseline was acquired for a minimum of 20 min at 50% maximum slope before high frequency stimulation (HFS; 2 × 100 Hz. 10 s interval) was applied. Responses were recorded for 60 min after HFS. Change in slope was calculated as a ratio of the average slope of the 20 min baseline (before HFS). Bar graphs of % fEPSP slope were calculated by averaging the last 5 min timepoints post HFS and normalized to baseline.

#### CaMKII binding to GluN2B *in vitro*

CaMKII/GluN2B binding assays were done as described.^6,13^ Briefly, GST-GluN2B-c-tail (GST-GluN2Bc; with Glun2B amino acids 1,120 to 1,480) was immobilized on anti-GST-antibody-coated microtiter plates (Thermo Scientific), blocked for 30 min with 5% BSA, and then overlaid for another 30 min at room temperature with 50 nM CaMKII (subunit concentration) in PIPES-buffered saline (pH 7.2) containing: 2 mM Ca^2+^, 1 μM CaM, 5 mM Mg^2+^, 0.1% BSA, 0.1% Tween 20. Inhibitors were added as indicated. After extensive washes in buffer containing 1 mM EGTA, GST-GluN2Bc and bound CaMKII was eluted for 10 min in SDS-loading buffer at 95°C. Bound CaMKII was measured via immunoblot.

#### Immunoblot analysis

Protein concentration was determined using the Pierce BCA protein assay (Thermo-Fisher). Before undergoing SDS-PAGE, samples were boiled in Laemmli sample buffer for 5 min at 95°C. Proteins were separated in a resolving phase polymerized from 10% acrylamide, then transferred to a polyvinylidene difluoride membrane at 24 V for 1–2 h at 4°C. Membranes were blocked in 5% milk or BSA and incubated with anti-CaMKIIα (1:2000, CBa2, available at Invitrogen but made in house), anti-CaMKIIa (1:2000, BD), anti-GST (1:2000, Millipore), followed by either Amersham ECL goat anti-mouse or anti-rabbit HRP-linked secondary 1:10000 (GE Healthcare). Blots were developed using chemiluminescence (Super Signal West Femto, Thermo-Fisher) imaged using the Chemi-Imager 4400 system (Alpha-Innotech), or imaged directly by fluorescence (Cytiva CyDye 700 goat anti-mouse and CyDye 800 goat anti-rabbit secondary antibodies, at 1:5000) using an OdysseyFc imaging instrument. All immunoblots were analyzed by densitometry (ImageJ; Version: 2.9.0/1.53t). Uncropped and unprocessed western blot images are provided in the Supplementary Information File.

#### *In vitro* phosphorylation assays

CaMKII activity was measured by ^32^P incorporation into the peptide substrate syntide 2, as described previously.^48^ Briefly, reactions were done for 1 min at 30°C and stopped by spotting the reaction mix onto P-81 phosphocellulose paper (Whatman), followed by extensive washing and scintillation counting. For measurement of stimulated activity in solution, the reaction mix contained 2.5 nM CaMKII (subunit concentration), 50 mM PIPES pH 7.1, 0.1% bovine serum albumine, 1 μM calmodulin, 2 mM CaCl_2_, 10 mM MgCl_2_, 75 μM syntide 2,100 μM [γ-^32^P]ATP (1 mCi/mmol) and inhibitor as indicated. For measurement of autonomous activity of GluN2B-bound CaMKII,^13^ the reaction mix contained 0.5 mM EGTA instead of calmodulin and CaCl_2_, 75 μM AC-2 as substrate peptide instead of syntide 2, and no soluble CaMKII was added. These latter reactions were carried out in the anti-GST-anti-body-coated microtiter plates after allowing CaMKII to bind to immobilized GST-GluN2Bc (as described above, including the wash with EGTA-containing buffer).

#### Cell culture of HEK293 cells

Human embryonic kidney cells (HEK293; authenticated STR analysis) were cultured in Dulbecco’s modified Eagle’s medium (DMEM; Gibco) supplemented with 10% fetal bovine serum (FBS; Sigma) and 1% penicillin/streptomycin solution (P/S; Gibco). HEK cells were not tested for mycoplasma. HEK cells were grown on 10 cm culture flasks and split every 3–4 days (at 90% confluency). For imaging experiments, cells were split into 12-well culture dishes on 18 mm No. 1 glass coverslips.

#### Live imaging of HEK293 cells

HEK cells were transfected with expression vectors for GFP-CaMKII mutants and pDisplay-mCh-GluN2B-c tail (2BC) and imaged as previously described.^44,49^ GFP-CaMKII colocalization with GluN2B in response to a Ca^2+^ stimulus induced by 10 μM ionomycin was monitored for 5–10 min at 32°C in imaging buffer (0.87x Hanks Balanced Salt Solution, 25 mM HEPES pH 7.4, 2 mM glucose, 2 mM CaCl_2_,1 mM MgCl_2_) by fluorescence microscopy. Images were acquired on a Zeiss Axiovert 200M equipped with a climate control chamber, using SlideBook software (Intelligent Imaging Innovations [3i]; Version: 6.0. Colocalization analysis was performed by calculating the Pearson’s correlation (correlation index) with ImageJ of pDisplay-mCh-2BC and GFP-CaMKII within the cytoplasm of HEK cells after background subtraction.

#### A cellular assay for CaMKII co-condensation with GluN2B

HEK cells were transfected and imaged as described above. However, in this case, GFP-CaMKII was co-expressed with a soluble, non-membrane targeted version of the mScarlet-GluN2B-c tail. Co-condensate formation was induced by triggering a Ca^2+^stimulus with 10 mM ionomycin for 6 min; dispersal or maintenance of the co-condensates was monitored after subsequent chelation of Ca^2+^ with 2.5 mM EGTA for an additional 5 min at 32°C in imaging buffer. HEK cells were pre-incubated for 15 min with 10 μM AS397 or Ruxolitinib where indicated. As with all constructs used here, the GFP coded for an EGFP with additional A206K mutation that minimizes dimerization, which is especially important for the condensation experiments.

#### Primary hippocampal culture preparation

To prepare primary rat hippocampal neurons, hippocampi were dissected from mixed sex rat pups (P0), dissociated in papain for 1 h, and plated at 100,000 cells/well on 18mm No. 1 glass coverslips in 12-well culture dishes for imaging in plating media: Minimal Essential Media (MEM, Gibco) containing 10% Fetal bovine serum (FBS, Sigma), 1% penicillin-streptomycin (P/S, Gibco). Plating media was replaced on DIV 1 with feeding media: Neurobasal A (NBA, Gibco) containing 2% B27 (Gibco) and 1% Glutamax (Sigma). On DIV 7, half of conditioned feeding media was replaced with fresh feeding media containing 2% 5-Fluoro-2′-deoxyuridine (FdU, Sigma). At DIV 14–18, neurons were transfected with 1 μg total cDNA per well using Lipofectamine 2000 (Invitrogen), then imaged 2–3 days later.

#### paCaMKII stimulation in hippocampal neurons

Neurons were wrapped in aluminum foil immediately following transfection and only exposed to red light to imaging. One image was then taken of each neuron to serve as a pre-photoactivation baseline. Immediately following this baseline image, paCaMKII was globally photoactivated with 405 nm laser pulse (100ms exposure, 75% laser power) once every 10 s, for a total of 60 s. Neurons were then imaged 15 min after stimulation and assessed for CaMKII synaptic enrichment and dendritic spine growth.

#### Image analysis of hippocampal neurons

DIV 15–18 neuronal cultures were transfected to express mCh-PSD-95 intrabody, iRFP cell fill, and GFP-paCaMKII T286A and imaged 24–48 h later. Images were collected at 32°C in HEPES buffered imaging solution containing (in mM) 130 NaCl, 5 KCl, 10 HEPES pH 7.4, 20 Glucose, 2 CaCl_2_, 1 MgCl_2_. Images of individual neurons from two independent cultures were acquired by 0.5 μm steps over 6 μm. 2D maximum intensity projection images were then generated and analyzed using a custom-build program in ImageJ. The program utilizes combinatorial thresholding to mask regions of the cell that contain high intensity PSD-95 puncta (the post-synaptic side of excitatory synapses in dendritic spines) and regions of the dendritic shaft that contain no fluorescence intensity of PSD-95. As a measure of synaptic enrichment, the ratio of mean CaMKII fluorescence intensity of the PSD-95 mask to the mean CaMKII fluorescence intensity in the dendritic shaft mask is measured. Spine growth was assessed by measuring the changes in mCherry cell fill fluorescence intensity within dendritic spine ROIs (F/F0).

### QUANTIFICATION AND STATISTICAL ANALYSIS

All data are shown as mean ± SEM. Statistical significance is indicated in the figure legends. Statistics were performed using Prism (GraphPad) software. Imaging experiments were obtained using SlideBook 6.0 software and analyzed using ImageJ. Immunoblots were analyzed by densitometry using ImageJ. All data were tested for their ability to meet parametric conditions, as evaluated by a Shapiro-Wilk test for normal distribution and a Brown-Forsythe test (3 or more groups) or an F-test (2 groups) to determine equal variance. All comparisons between two groups met parametric criteria, and independent samples were analyzed using unpaired student’s t-tests. Comparisons between three or more groups meeting parametric criteria were done by one- or two-way ANOVA with specific post-hoc analysis indicated in figure legends. Sample sizes for this study were determined based on previous experience; post-hoc power analysis was conducted, confirming that our studies were adequately powered to detect statistical significance of effects. Biological replication was achieved by measuring each unique cell, sample, and hippocampal slice once. Cells were derived from at least two separate cultures and a minimum of four distinct wells and slices were derived from at least 3 individual animals per group. Randomization was accomplished by reversing the sample order for every experiment. Investigators were not blinded to the samples during collection or analysis. Blinding was not performed due to resources constraints combined with the nature of experiments and analysis having low potential for introducing bias.

## Supplementary Material

1

## Figures and Tables

**Table:** KEY RESOURCES TABLE

REAGENT or RESOURCE	SOURCE	IDENTIFIER
Antibodies		
Anti-CaMKII antibody	In house	CBα2; RRID: AB_2533032
Anti-GST antibody	Millipore	AB3282; RRID: AB_91439
CyDye 800 goat anti-rabbit	Cytiva	Product #: 29360791
CyDye 700 goat anti-mouse	Cytiva	Product #: 29360785
Bacterial and virus strains		
Baculovirus/Sf9 cell system	Thermo Fisher Scientific	RRID: CVCL_0549
BL21(DE3) Competent E.coli	Invitrogen	Product #: EC0114
Chemicals, peptides, and recombinant proteins		
Ionomycin, Calcium Salt	Thermo Fischer Scientific	Catalog #: I24222
Calcium Phosphate	Thermo Fischer Scientific	Catalog #: 446390010
Reduced glutathione	Sigma Aldrich	Product #: G4251
IPTG (Isopropyl β-D-1-thiogalactopyranoside)	Sigma Aldrich	Product #: I5502
Ammonium sulfate	Sigma Aldrich	Product #: 31119
NaCl (Sodium chloride)	Sigma	Catalog #: S5886
Calcium chloride (CaCl_2_)	Sigma	Catalog #: C7902
Magnesium chloride (MgCl_2_)	Sigma	Catalog #: M1828
Adenosine diphosphate (ADP)	Millipore Sigma	Product #: A5285
Adenosine triphosphate (ATP)	Sigma	Product #: A2383
EGTA (ethylene glycol-bis(β-aminoethyl ether)-N,N,N′,N′-tetraacetic acid)	Sigma Aldrich	Product #: E4378
EDTA (Ethylenediamine tetraacetic acid)	Sigma Aldrich	Product #: 324503
Tween 20	Sigma	Product #: P7949
BSA (Bovine Serum Albumin)	Sigma Aldrich	Product #: A4612
PIPES1,4-Piperazinediethanesulfonic acid)	Sigma Aldrich	Product #: P1851
HEPES (N-2-hydroxyethylpiperazine-N-2-ethane sulfonic acid)	Gibco	Catalog #: 15630080
PBS (PIPES-buffered saline), pH 7.2	Gibco	Catalog #: 20012050
lysozyme	Millipore Sigma	Product #: 1.05281
Tris base	RPI	Product #: T60040
ribonuclease A	Sigma Aldrich	Product #: R6513
deoxyribonuclease I	Thermo Scientific	Catalog #: 89836
β-mercaptoethanol	Sigma	Catalog #: M6250
Glucose	Sigma Aldrich	Product #: G7021
0.87x Hanks Balanced Salt Solution	Sigma Aldrich	Product #: H6648
AS397	Collaborator	Not Available
AS283	Collaborator	Not Available
AS105	Collaborator	Not Available
AIP	Anaspec	Catalog #: AS-64929
Ruxolitinib	MedChem Express	HY-50858
Tofacitinib	MedChem Express	Cat3 hy-40354
KN93	Tocris	Catalog #: 5215
tatCN21	Chi Scientific	N/A (custom peptide)
complete protease inhibitor cocktail (EDTA-free)	Roche	–
Experimental models: Cell lines		
HEK293 cells	ATCC	RRID: CVCL_0045
Software and algorithms		
Prism 10	Graphpad	RRID:SCR_002798
ImageJ	NIH	RRID:SCR_003070
Slidebook 6.0	Intelligent Imaging Innovations (3i)	RRID:SCR_014300
Other		
Dulbecco’s Modified Eagle Medium (DMEM)	Gibco	Catalog #11965-092
Fetal Bovine Serum (FBS)	Sigma	Catalog #F0926
Penicillin/Streptomycin solution	Gibco	Catalog #11965-092
CaM-Sepharose beads	GE Healthcare	Product #: GE17-0529-01
Phospho-cellulose column	Whatman	Product #: P7892
Phenyl-Sepharose column	Sigma	Catalog #: A32562
Glutathione Sepherose 4B beads	Cytiva	Product #: 17075601
4–20% Mini-PROTEAN^®^ TGX^™^ Precast Protein Gels	Bio Rad	Product #: 4561094
GST-GluN2B fusion proteins	Made in house (lab)	GST-GluN2Bc
Pierce^™^ Anti-GST Coated Plates	Thermo Fischer Scientific	Catalog #: 15144
Minimal Essential Media (MEM)	Gibco	Catalog #: 11090-081
Neurobasal A (NBA)	Gibco	Catalog #: 10888-022
B27	Gibco	Catalog #: 17504-044
Glutamax	Gibco	Catalog #: 35050-061
5-Fluoro-2′-deoxyuridine	Sigma	Catalog #: F-0503
Lipofectamine 2000	Invitrogen	Catalog #: 52887
Syntide-2	Genscript	Catalog #: RP10322
AC-2	Genscript	Catalog #: RP20277
ATP γ-P32	Perkin-Elmer	NEG035C001MC
CaMKIIα	Made in House	Not Available
Calmodulin	Made in House	Not Available

## Highlights

- GluN2B binding is the structural CaMKII function required for LTP induction
- Autonomous activity of GluN2B-bound CaMKII mediates LTP expression
- Enzymatic CaMKII activity is not required for LTP maintenance
- CaMKII pT286 is not required for LTP expression or maintenance
