# Supplementary material for: LTP expression mediated by autonomous activity of GluN2B-bound CaMKII
Source: Cell Rep. Author manuscript; Available in PMC 2024 Nov 14. (PMC11563194; doi:10.1016/j.celrep.2024.114866)
Supplement: 1 [file NIHMS2031323-supplement-1.pdf]

Cell Reports, Volume *43*

Supplemental information

LTP expression mediated by autonomous activity of GluN2B-bound CaMKII

Nicole L. Rumian, C. Madison Barker, Matthew E. Larsen, Jonathan E. Tullis, Ronald K. Freund, Amir Taslimi, Steven J. Coultrap, Chandra L. Tucker, Mark L. Dell'Acqua, and K. Ulrich Bayer

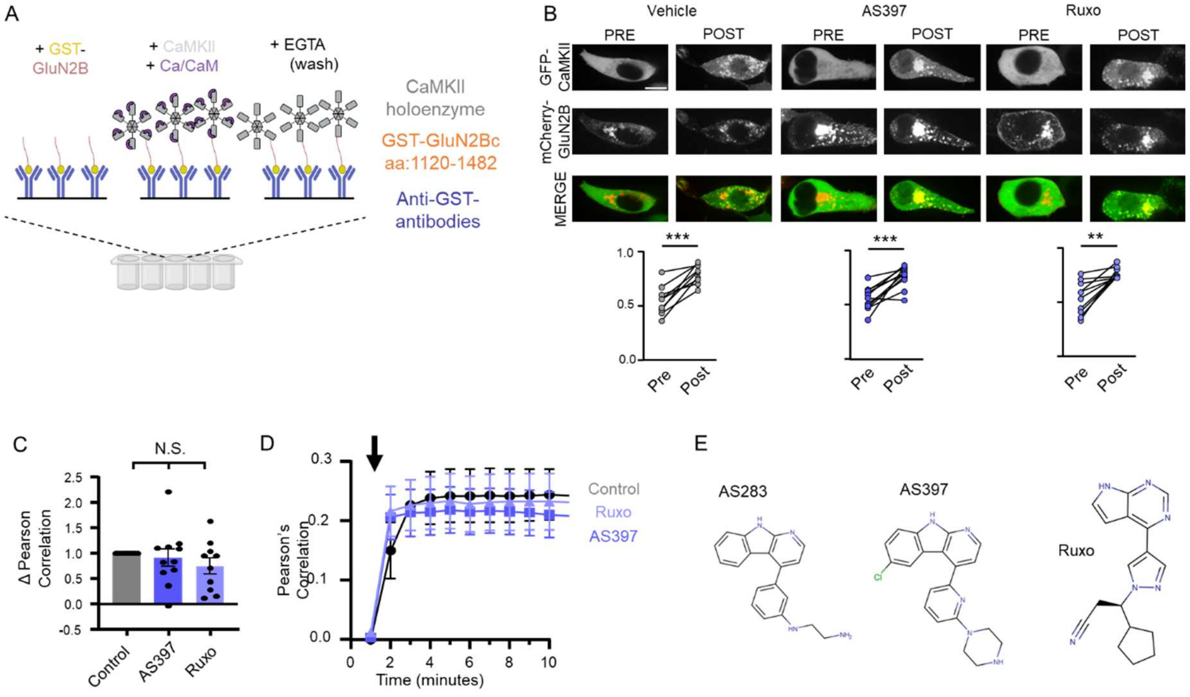

Figure S1. Effects of AS397 and Ruxolitinib on CaMKIIα binding to GluN2Bc, Related to Figure 1. (**A**) Schematic illustration of the *in vitro* assay for CaMKII binding to GluN2B. Briefly, GST-GluN2Bc was immobilized on anti-GST-antibody coated microtiter plates. CaMKIIα binding was stimulated by Ca^{2+}/CaM in absence of nucleotide but in presence of ATP-competitive inhibitors as indicated. Then, the plates were washed with EGTA containing buffer, the bound CaMKII was eluted in SDS-loading buffer at 95°C, and probed by immunoblot. (**B**) CaMKII binding to GluN2B was examined in HEK cells by colocalization of GFP-CaMKIIα (green) with a membrane targeted mCherry-GluN2Bc (red) in HEK 293 cells. Binding is promoted by ionomycin-induced Ca^{2+} signals. Shown are images before and 10 min after ionomycine treatment, with prior 15 minutes pre-incubation with 10 µM AS397 or Ruxo as indicated. Ionomycine induced a significant increase in co-localization in all conditions (****P*<0.001, ***P*<0.01, paired t-test). Scale bar, 10 µm. (**C**) The net change in CaMKIIα/GluN2B co-localization in the experiment described in panel B was the same for all conditions tested (N.S., one-way ANOVA). (**D**) Time-lapse analysis of CaMKIIα co-localization with GluN2B in HEK cells. The black arrow indicates ionomycin treatment. (**E**) Structures of AS283, AS397 and Ruxolitintib.

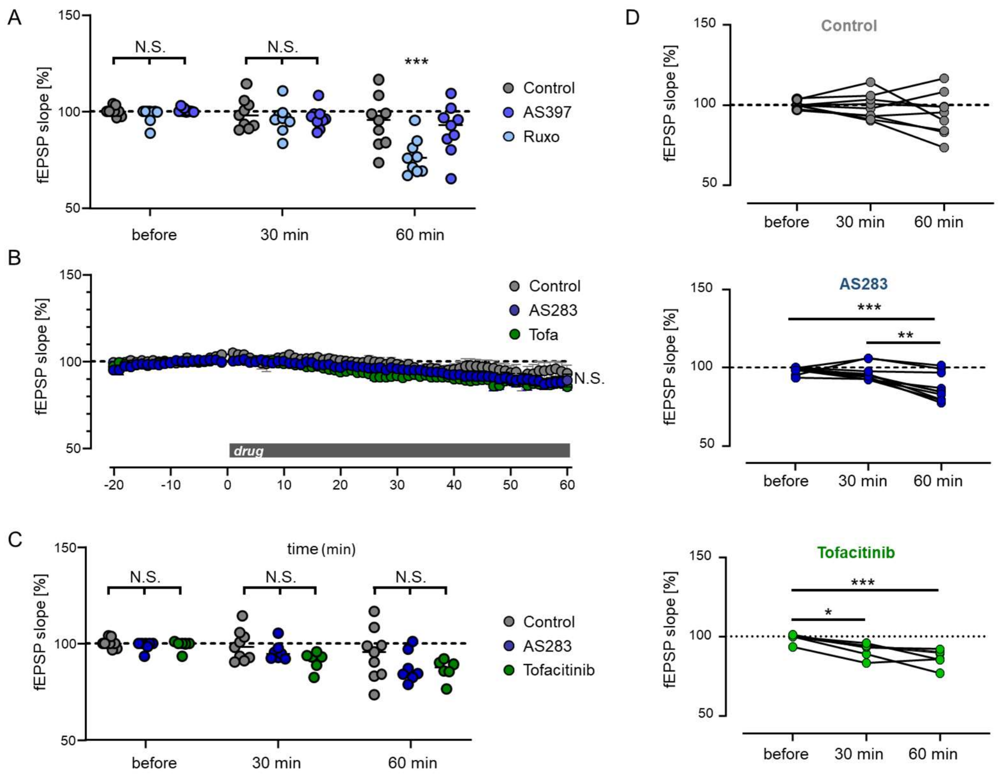

Figure S2. Effect of ATP-competitive CaMKII inhibitors on basal synaptic transmission, Related to Figure 2. (**A**) Individual fEPSP slope data from slices treated with no drug (Control), AS397 or ruxolitinib (Ruxo) during a baseline period (before), at 30 min after drug application or at 60 min drug after drug application (*n* = 9 for all conditions). There were no significant differences between groups during the before or 30 min period, but Ruxo was significantly reduced compared to control at 60 minutes (*** *P*< 0.001 by one-way ANOVA followed by Bonferroni's post-hoc analysis). (**B**) The mild fEPSP decrease after addition of AS283 or tofacitinib (*n* = 7 and 6 slices, respectively) was not significantly different compared to the control condition also shown in Figure 2C (although significance was reached for both drugs compared to their baseline; see panel D). (**C**) Individual fEPSP slope data from slices treated with no drug (Control *n* = 9), AS283 (*n*=8) or Tofacitinib (*n*=7) during a baseline period (before), 30 min after drug application or 60 min after drug application. There were no significant differences between groups for any of the time points (one-way ANOVA). (**D**) Individual fEPSP slope data from slices treated with no drug (Control, top *n* = 9) AS283 (middle; *n* = 8) or Tofacitinib (bottom; *n* = 7) during a baseline period (before), 30 min or 60 min after drug addittion. * *P*< 0.05, ** *P*< 0.01, *** *P*< 0.001 by repeated measures one-way ANOVA followed by Bonferroni's post-hoc analysis.

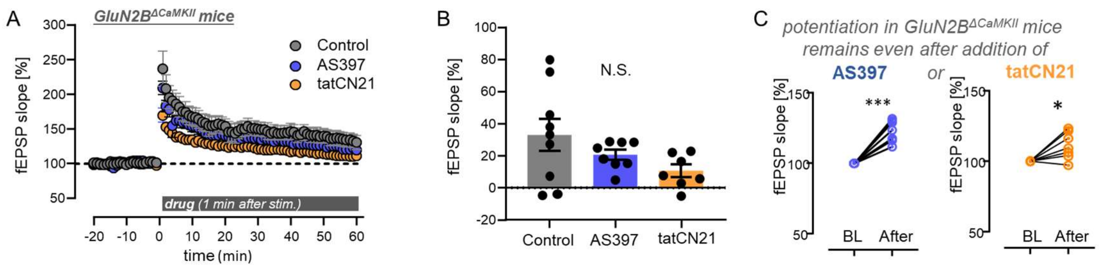

Figure S3. LTP in slices from GluN2B$^{\Delta CaMKII}$ mice: effect of post-induction addition of CaMKII inhibitors, Related to Figure 4. LTP was induced by 2x HFS and measured by the fEPSP slopes at the CA3 to CA synapses in hippocampal slices from mice with the GluN2B$^{\Delta CaMKII}$ mutant that prevents CaMKII binding. AS397 (10 µM) or tatCN21 (5 µM) was added 1 min after LTP induction. Data show mean ± S.E.M. (**A**) When added 1 min after LTP induction, tatCN21 or AS397 (two CaMKII inhibitors that completely reverse LTP when added at that time point; see Figure 3) appeared to show only a mild reduction in fEPSP slope when compared to control without drug also shown in Figure 4A,B (*n* = 7 and 8 slices for tatCN21 and AS397, respectively). (**B**) During the last 5 min of recordings, the apparent reduction of fEPSP slope after tatCN21 or AS397 addition as in panel C was not significant compared to control without drug. (**C**) When comparing baseline period (BL) to 60 minutes after LTP induction (After), significant potentiation over baseline remained in slices from GluN2B$^{\Delta CaMKII}$ mice even after addition of AS397 or tatCN21 (* $P < 0.05$, *** $P < 0.001$ by two-tailed paired *t*-test). By contrast, slices from wild type mice, the same treatments completely blocked any LTP assessed in the same manner (see Figure 3).

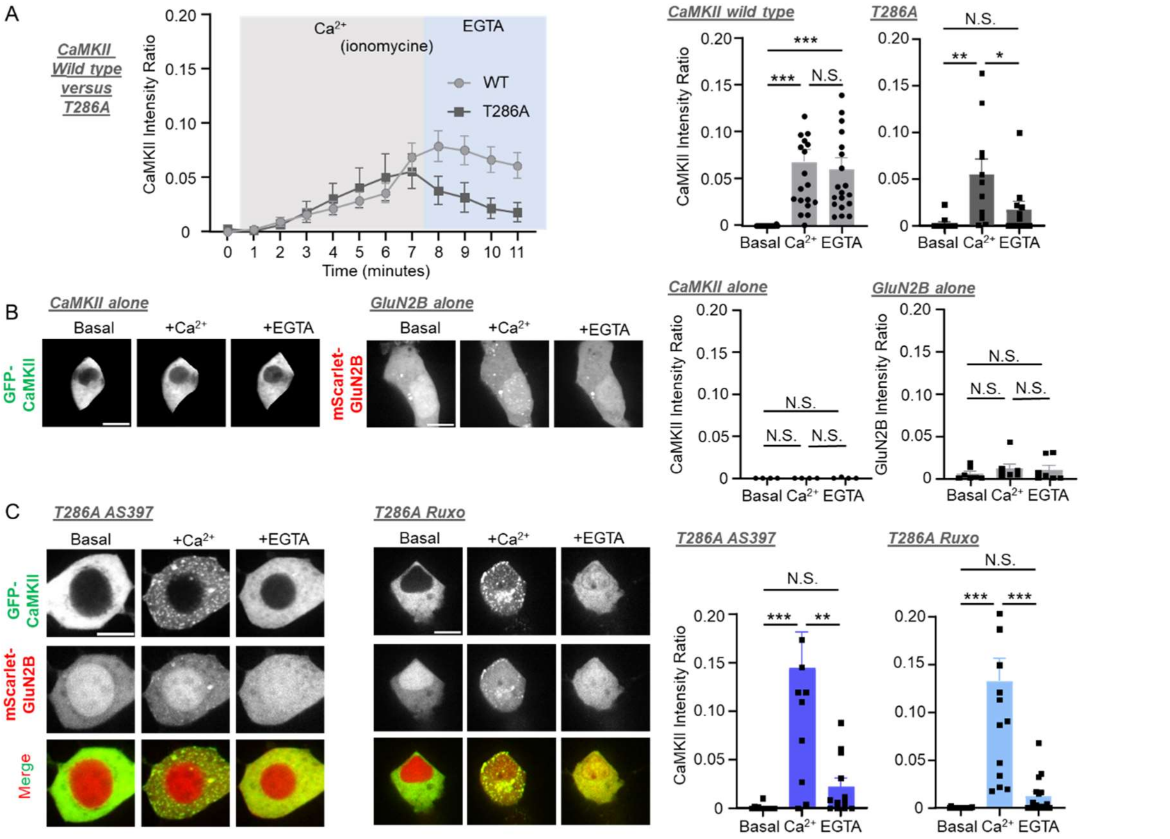

Figure S4. CaMKII co-condensation with GluN2B in HEK cells, Related to Figure 4. Scale bars, 10 µm. N.S., not significant; *P< 0.05; **P < 0.01; ***P < 0.001; in one-way ANOVA with Tukey's posthoc analysis. (**A**) Quantification of the co-condensation of GFP-CaMKII and mScarlet-GluN2B-c seen in Fig. 4C in HEK cells after a Ca^{2+} stimulus with ionomycin. Left panel: time course; right panels: bar graphs of last images before stimulation, last image before Ca^{2+} chelation of with EGTA, and last image after the addition of EGTA. The Ca^{2+}-induced cluster formation is maintained after EGTA addition only for CaMKII wild type, but not for the T286A mutant. (**B**) When expressed individually, neither GFP-CaMKII nor mScarlet-GluN2B-c form significant clusters in HEK cells upon a Ca^{2+} stimulus with ionomycin. For GluN2B, some level of basal clusters is observed. For CaMKII, previously described cluster formation in response to ionomycin additionally requires lowering the pH. (**C**) The co-clustering of the CaMKII T286A mutant with GluN2B after a Ca^{2+} stimulus is reversed by chelating Ca^{2+} with EGTA even in the presence of the ATP-competitive CaMKII inhibitors AS397 or ruxolitinib (10 µM) that rescued LTP in the T286A mutant hippocampal slices (see Figure 1A,B), indicating that rescue of LTP maintenance is not mediated by rescue of maintenance of co-condensation.
